# *Operando* anomalous X-ray powder diffraction interleaved with X-ray absorption spectroscopy using a scanning 2D imaging detector on the XMaS beamline: design, implementation and performance

**DOI:** 10.1107/S1600576725007022

**Published:** 2025-09-18

**Authors:** Dariusz Wardecki, Paul B. J. Thompson, Kinga Mlekodaj, Mark G. Dowsett, Mieke Adriaens, Amy J. Knorpp, Catherine Dejoie, Kinga Góra-Marek, Jeroen A. van Bokhoven, Mark A. Newton, Przemyslaw Rzepka

**Affiliations:** a Faculty of Physics University of Warsaw Pasteura 5 Warsaw02-193 Poland; b Department of Structure and Dynamics in Catalysis, J. Heyrovský Institute of Physical Chemistry, Czech Academy of Sciences, Dolejškova 2155/3, Prague182 23, Czechia; c XMaS beamline, UK CRG European Synchrotron Radiation Facility 71 avenue des Martyrs CS 40220 Grenoble Rhône-Alpes 38043 France; d Department of Physics University of Warwick Coventry WarwickshireCV4 7AL United Kingdom; e Department of Chemistry Ghent University Krijgslaan 281-S12 GhentB-9000 Belgium; f Chemical Energy Carriers and Vehicle Systems Laboratory Swiss Federal Laboratories for Materials Science and Technology Überlandstrasse 129 DübendorfCH-8600 Switzerland; g ID22 European Synchrotron Radiation Facility 71, avenue des Martyrs CS 40220 Grenoble Rhône-Alpes 38043 France; h Faculty of Chemistry Jagiellonian University Gronostajowa 2 Krakow30-387 Poland; i Institute for Chemical and Bioengineering ETH Zürich Vladimir-Prelog-Weg 1-5/10 Zürich8093 Switzerland; j Center for Energy and Environmental Sciences Paul Scherrer Institute Villigen5232 Switzerland; Australian Synchrotron, ANSTO, Australia

**Keywords:** scanning 2D imaging detectors, anomalous X-ray powder diffraction, AXRPD, transmission X-ray absorption spectroscopy, XAS, heterogeneous catalysis, zeolites

## Abstract

We present a method for achieving high-quality anomalous X-ray powder diffraction, interleaved with transmission X-ray absorption spectroscopy, using a flat-panel imaging detector scanned over a large angular range. Anomalous X-ray powder diffraction is a technique that enables the highlighting of specific elements within a crystal structure and pinpointing the nature of active (or inactive) sites involved in specific reactions *in situ* or in an *operando* manner. This approach enables the collection of a *Q* range that is not attainable using static flat-panel detectors in the absorption energy regimes used (8.94–17.5 keV). We consider the advantages and limitations of such an approach compared with alternative methods and describe in detail how it is achieved, along with the data processing and workflow required for an accurate restoration of conventional X-ray powder diffraction and anomalous X-ray powder diffraction data from sequentially acquired images. We demonstrate the applicability and capacity of this method for the *in situ*/*operando* restoration of the positions and atomic arrangement of copper atoms in the elevated-temperature aerobic activation of a copper-ion-exchanged zeolite, Cu–mazzite.

## Abstract 2

A method is presented for achieving high-quality anomalous X-ray powder diffraction with an extended *Q* range using a flat-panel imaging detector that scans over a large angular range, coupled with transmission X-ray absorption spectroscopy. When combined with the *in situ* sample environment, this approach enables the isolation of specific elements within long-range and local structures, while tracking their evolution during reactions.

## 1. Introduction

The ability to describe the spatial arrangement of atoms with atomic resolution within functional materials, and how those atoms might change their disposition during reaction, is highly sought after in many areas of research. X-ray powder diffraction (XRPD) is one technique by which such ends may be achieved, especially when it is exploited at an atomic X-ray absorption edge (called anomalous or resonant X-ray powder diffraction, AXRPD) to highlight the crystallographic position of a specific element in a structure [see, for example Wilkinson *et al.* (1992 ▸), Nishihata *et al.* (2002 ▸), Palancher *et al.* (2012 ▸) and Pinar *et al.* (2021 ▸)].

AXRPD exploits energy-dependent variations in X-ray scattering to enhance the contrast of specific atomic species within a structure. In this technique, the incident X-ray energy is adjusted to lie near or away from an absorption edge of the element of interest. The atomic scattering factor *f* = *f*_o_ + *f*_1_ + *if*_2_ primarily depends on the number of electrons (*f*_o_), but at near-resonance conditions the complex terms (*f*_1_ + *if*_2_) become significant, selectively influencing the scattering from resonant scatterers while leaving other elements largely unaffected. AXRPD has been applied to identify elements with absorption edges suitable for diffraction studies, including Br (13.5 keV), Rb (15.2 keV), Se (12.66 keV), Zn (9.66 keV) and Fe (7.11 keV) (Cromer & Liberman, 1970 ▸; Grenier & Joly, 2014 ▸; Hendrickson, 2014 ▸; Finkelstein *et al.*, 1992 ▸), and a similar approach has been used in multi-wavelength anomalous diffraction to enhance structural studies of biological macromolecules (Hendrickson, 1991 ▸). In zeolite frameworks, AXRPD has been instrumental in highlighting heteroatom distributions, identifying the nature of Ti active sites in TS-1 catalysts, (Rzepka *et al.*, 2024 ▸), and pinpointing Al locations in FER (Pinar *et al.*, 2021 ▸) and ZSM-5 zeolite structures (Rzepka *et al.*, 2025 ▸; Li *et al.*, 2025 ▸). In the latter, AXRPD revealed the ordering of isolated Al atoms and Al pairs [Al–(SiO_2_)_1–2_–Al sequences], providing critical insights into ZSM-5 framework organization. The method, when applied at the Cu *K* edge, isolates the signal scattered by copper from that generated by water and extra-framework oxygen present within the structures of Cu zeolites. This approach was successfully used in an *ex situ* manner to highlight the structure of paired Cu monomers in copper-exchanged mazzite (Knorpp *et al.*, 2021 ▸) and then latterly track their evolution during the MtM reaction (Wieser *et al.*, 2024 ▸). These studies underscore AXRPD as a powerful tool for refining zeolite structural models and pinpointing active sites critical to catalytic performance.

Nevertheless, AXRPD is an extremely demanding technique in a number of ways. If advanced crystallographic data analysis is to be applied successfully, high-resolution data are required, acquired reproducibly over a large *Q* (2θ) range [*Q* = (4π/λ) sin θ, where 2θ is the scattering angle and λ is the wavelength of the incident radiation] and with high signal-to-noise ratio, which places a heavy burden on both the quality of the source and the means of detection. These requirements also place a number of restrictions upon the presentation of the sample and the sample environment, especially when considering powder samples studied *in situ* within capillary-type plug-flow reactors in reactive environments at variable temperatures. Performing a successful experiment, with proper gas flow and contact with the powder, at the same time as achieving the required quality of data, is not trivial. The projection of the sample’s thickness onto the detected X-ray scattering must be carefully monitored, as this can easily compromise the achievable resolution by causing diffraction peak broadening. This often necessitates the use of very thin capillaries (≪1 mm) to limit signal attenuation due to absorption. However, it is crucial to maintain a large number of randomly oriented crystallites within the diffraction volume to enhance statistical reliability and minimize the graininess effect. Samples composed of heavily absorbing elements may also require additional dilution to reduce overall X-ray absorption, though, as has been demonstrated (Grunwaldt *et al.*, 2004 ▸), the diluent itself can have adverse effects on other aspects of the experiment and must be previously established to be chemically inert under the conditions to be investigated.

Specimen displacement, which can easily occur in variable-temperature experiments, also needs to be avoided for the accurate measurement of the Bragg peak positions in crystallographic data. Lastly, prefered orientation of crystallites within the powder sample needs to be either suppressed or accounted for. For *ex situ* and static studies, this latter problem can be partially addressed by spinning the sample to compensate for the over-represented orientations in the axial plane of the diffraction measurement geometry. However, accounting for the equatorial planes, such as when crystals lie along the capillary, is much more challenging. Moreover, spinning the capillary through 180° may be difficult or impossible if the measurements require gas or liquid flow through the sample, and ‘wiggling’ or slow rotation of the reactor may be insufficient (Agostini *et al.*, 2010 ▸; Wragg *et al.*, 2021 ▸).

The requirement for very thin samples also has consequences for various aspects of the *operando* experiment itself. For instance, the precise measurement of temperature, essential when considering the evolution of a process or a material [see *e.g.* Newton *et al.* (2019 ▸)], becomes more difficult to achieve, and a direct method of temperature measurement (such as a thermocouple inserted directly into the thin sample bed) is often not a viable option. Moreover, the correct contact of the medium (be it a gas or liquid) with the sample – which is to say, in catalysis, the achievement of plug flow – becomes much harder to guarantee within such small reactors and with densely packed materials. Lastly, the sample environ­ment itself must be arranged to allow the unencumbered collection of X-ray scattering data over an angular range sufficient to obtain the *Q* (2θ) space required for high-level analysis by means of, for instance, pair distribution function (calculated from data collected at higher energies) or Rietveld analysis methods.

Maintaining an adequate *Q* range becomes particularly challenging in experiments involving the collection of AXRPD data at atomic absorption edges which are often found at relatively low energies.

Nevertheless, solutions to these issues have been proposed and validated for a variety of applications [see *e.g.* Newton *et al.* (2019 ▸), Clausen *et al.* (1993 ▸), Chupas *et al.* (2008 ▸), Jensen *et al.* (2010 ▸) and Marshall *et al.* (2023 ▸)]. However, it remains the case that a great deal of care and attention is required to attain the sorts of *operando* XRPD that can be used to derive the crystal structure of working powder materials with high accuracy.

Broadly speaking, for the collection of X-ray scattering data, three solutions, each of which comes with its own strengths and drawbacks, currently prevail: static 2D flat-panel detection systems (He, 2018 ▸); arc detectors (Evain *et al.*, 1993 ▸), which can instantaneously capture the required angular range of detection with the required resolution; or multi-crystal analyser stages that may be scanned over the required angular range (Hodeau *et al.*, 1998 ▸). All are operated in transmission geometry, which utilizes a near-parallel incident beam of X-rays with sufficient cross section to illuminate the entire powder sample.

Flat-panel detectors capture diffracted signals in transmission geometry, enabling textural assessments. They offer fast acquisition but require post-processing due to resolution limits (Donath *et al.*, 2013 ▸), which often requires the use of a dedicated resolution function (Chernyshov *et al.*, 2021 ▸). The Pilatus3 R 300K, a direct conversion detector, minimizes noise, supports high-speed readout and handles intense X-ray fluxes, providing a large active area for detailed diffraction analysis.

The Pilatus detector uses a silicon sensor. Each pixel is equipped with a 20-bit counter that can count up to 1048574 counts per pixel, which essentially defines its dynamic range.

The detector’s linear range is inherently limited by its dead time τ. Because each detection event is followed by a short dead time (typically of the order of 50–250 ns), the detector can only respond linearly to incident photon fluxes that yield count rates well below the maximum count rate of roughly 1/τ per pixel. When the photon flux becomes too high, pile-up and saturation effects cause the measured count rate to deviate from linearity, and offline corrections [using, for example, the relation *N*_obs_ = *N*_o_ exp(−*N*_o_τ)] must be applied to retrieve the true photon flux. This interplay between the dead time and the maximum count capacity governs the available photon flux range over which the detector maintains a linear response. The Pilatus detector performs very well in the range of a few hundred thousand to about 1 × 10^6^ photons per second per pixel. For example, at a 50% relative global threshold the measurements show that, with low gain settings, the detector is accurate (with only about a 2% error) at an incident rate of roughly 420000 photons per second per pixel and, with medium gain settings, at around 630000 photons per second per pixel. Deviations from the expected behaviour (due to pile-up effects) become significant when the incident rate exceeds about 1 × 10^6^ photons per second per pixel (Kraft *et al.*, 2009 ▸).

Other solutions for collecting X-ray scattering include arcuate strip detectors (Evain *et al.*, 1993 ▸) and multi-crystal analyser stages (Hodeau *et al.*, 1998 ▸). These systems naturally provide both the angular range and accuracy required for such investigations, allowing for rapid data collection over a large angular range without the need for physical movement of the detector. However, one drawback of these detector systems is their limited ability to address preferred orientations. To mitigate this issue, the sample must be spun or rocked over a small angular range.

One effective solution to meet all the requirements described above is the use of a multi-crystal analyser stage combined with a 2D pixel detector, as implemented on the high-resolution powder diffraction beamline ID22 at the European Synchrotron Radiation Facility (ESRF) in Grenoble, France (Fitch *et al.*, 2023 ▸). In this setup, the diffracted photons must satisfy the Bragg condition imposed by the analyser crystals, resulting in narrow and well defined peak shapes and positions. Unlike conventional setups, the use of a thin capillary to avoid peak broadening is not a requirement (other than for controlling absorption). Additionally, the analyser crystals make peak positions insensitive to displacement-type aberrations and efficiently reduce parasitic scattering from air or sample environments, as well as fluorescence from the sample.

The integration of a 2D detector positioned behind the analyser crystals provides further advantages, such as the correction of low-angle axial divergence (peak asymmetry), improvement of the statistical quality of high-angle data (Fitch & Dejoie, 2021 ▸) and mitigation of graininess effects (Dejoie *et al.*, 2018 ▸). As a result, high-resolution powder diffraction patterns can be collected within minutes across an energy range of 6–75 keV, making the setup highly effective for a wide variety of *in situ* and *operando* measurements. However, due to the high photon selectivity of the method, a high flux is required, which is provided on ID22 by an undulator source coupled with the new EBS ring.

Here, we present an alternative solution for obtaining XRPD data from an *operando* experiment, based on a concept previously described by Dowsett *et al.* (2020 ▸). In this approach, a flat-panel 2D detector is incrementally stepped through the required 2θ range in small angular steps. This method offers several advantages, including a large *Q* range, high dynamic range, excellent angular resolution, high sensitivity and statistical precision, and the ability to correlate sample texture with features in the resulting 1D pattern extracted from the data. Diffraction images acquired at each angular increment are transformed and merged into a single image covering the whole angular range and the 1D pattern is integrated out of this. The data processing uses the software package *esaProject* (developed by EVA Surface Analysis; available on request from M. Dowsett: markdowsett.esa@gmail.com). We augment the method by interleaving XAS measurements with the image acquisition and by making resonant scattering measurements. Whilst the use of scanning 2D detectors is not unknown, and similar arrangements can be found both in commercial laboratory instruments (He, 2018 ▸) and on some existing beamlines (Abdellatief *et al.*, 2022 ▸; Fitch *et al.*, 2023 ▸; Kieffer *et al.*, 2020 ▸), they have not previously been demonstrated for *operando* crystallography using gas-flow reactor systems for catalytic applications, as far as we are aware.

This approach, when combined with successfully implemented interleaved X-ray absorption spectroscopy (XAS) measurements, becomes particularly valuable as conventional XRPD provides no direct information about the chemical state of the elements in the materials under study. We show that the combination of XAS, XRPD and resonant scattering (AXRPD) methods described here permits the *operando* study of the evolution of chemical speciation alongside structure. XAS also provides a means to verify, to some degree, that the chemistry itself has not been compromised by any unfortunate (and maybe not so evident) failures in any aspect of the applied experimental protocol, or indeed the application of the X-rays themselves (Newton *et al.*, 2020*a* ▸; Bras *et al.*, 2022 ▸; Thomä & Zobel, 2023 ▸). XAS, and spectroscopy in general, are very well adapted to interrogate this aspect of materials chemistry and therefore guard against the derivation of erroneous conclusions that could result from the application of diffraction alone.

In the following, we outline this combined approach to *in situ* AXRPD-based crystallography and XAS, including the physical setup, the sample environments used and the experimental method. We summarize the strengths and weakness associated with them and the data processing workflow required to restore the data to be submitted to analysis. Finally, we demonstrate that this approach, using both monochromatic and anomalous (resonant) powder diffraction approaches, does indeed result in a precise crystallographic description of the speciation and location of copper in the example material, mazzite. Through this demonstration, regarding the activation of Cu–mazzite (Cu–MAZ) in respect of the direct and selective conversion of methane to methanol (MtM) (Groothaert *et al.*, 2005 ▸; Newton *et al.*, 2020*b* ▸; Knorpp *et al.*, 2018 ▸; Knorpp *et al.*, 2019 ▸; Wieser *et al.*, 2023 ▸; Wieser *et al.*, 2024 ▸), we offer a new solution for this type of *operando* study, from which a more profound understanding of how materials work may be derived.

## 2. Experimental details

### 2.1. Selection of the Cu–MAZ system in respect of the demonstration of the protocol

We begin with a brief description of the system chemistry and what is known regarding it. The direct and selective oxidation of methane to methanol using copper-exchanged zeolites and either stepwise or catalytic approaches has been heavily investigated since its discovery in 2005 (Groothaert *et al.*, 2005 ▸). This chemistry could potentially be utilized to develop processes that can be applied in remote locations and at a reduced scale, to take methane, which would otherwise be flamed off (to yield CO_2_), and convert it into a useful resource in an economically viable, sustainable and otherwise environmentally friendly manner. Since 2005 many different Cu–zeolite systems have been investigated, and significant advances in the understanding and performance of these materials for this chemistry have been realized (Newton *et al.*, 2020*b* ▸). Whilst an economically viable process founded upon these materials has yet to appear, Cu–MAZ has recently emerged (Knorpp *et al.*, 2018 ▸; Knorpp *et al.*, 2019 ▸; Wieser *et al.*, 2023 ▸) as, by some margin, the best Cu–zeolite system thus far demonstrated for this conversion, not just in terms of single-pass yields and selectivity but also in terms of the far more important, though far more rarely considered, metric of process productivity (Wieser *et al.*, 2023 ▸).

The atomic positions of the copper atoms within the Cu-MAZ crystal structure that result from the activation of the system in O_2_ up to 723 K have been established through the application of AXRPD (Knorpp *et al.*, 2021 ▸). However, these measurements were made *ex situ* at ambient temperature after the reaction was completed. As a result, whilst the actual active (and, indeed, inactive) copper sites are well described for this system, their *operando* evolution, whose understanding is important for process optimization, is not.

There have been numerous previous *operando* studies (Vanelderen *et al.*, 2015 ▸; Liu *et al.*, 2017 ▸; Pham *et al.*, 2014 ▸; Knorpp *et al.*, 2021 ▸; Sushkevich *et al.*, 2017 ▸; Brenig *et al.*, 2024 ▸; Wieser *et al.*, 2024 ▸; Pappas *et al.*, 2017 ▸) focusing on various Cu–zeolite systems since 2004. However, these studies have predominantly relied on methods such as XAS, UV–Vis and IR spectroscopies, and electron paramagnetic resonance, which by their nature cannot precisely determine, in three dimensions, the exact location of active Cu atoms at any given moment, or their spatial relationships or possible changes in siting under reaction conditions. While these techniques have provided valuable insights, they have not been able to define unambiguously the true nature of the active sites at any point in time in a direct and explicit way.

The only current exception is the Cu–MAZ system, where APXRD was first applied *ex situ* (Knorpp *et al.*, 2021 ▸) and subsequently in an *operando* manner (Wieser *et al.*, 2024 ▸). These studies demonstrated that Cu–MAZ contains a single active site composed of two Cu atoms arranged as proximal monomers that cooperate in facilitating the two-electron transfer required for the conversion of methane to a meth­oxy species. Consequently, the Cu–MAZ system serves as an ideal model for demonstrating and validating the novel approach to *operando* AXRPD that we are currently developing.

### 2.2. Experimental arrangement

Both the XRPD and XAS measurements were performed on BM28 (XMaS, UK CRG beamline) at the ESRF. The conceptual design of the beamline optics, originally proposed in the mid-1990s (Paul *et al.*, 1995 ▸), yields a beamline that is simple to use and which now maximizes the flux from the ESRF short dipole source (Thompson *et al.*, 2019 ▸).

**Figure 1:**
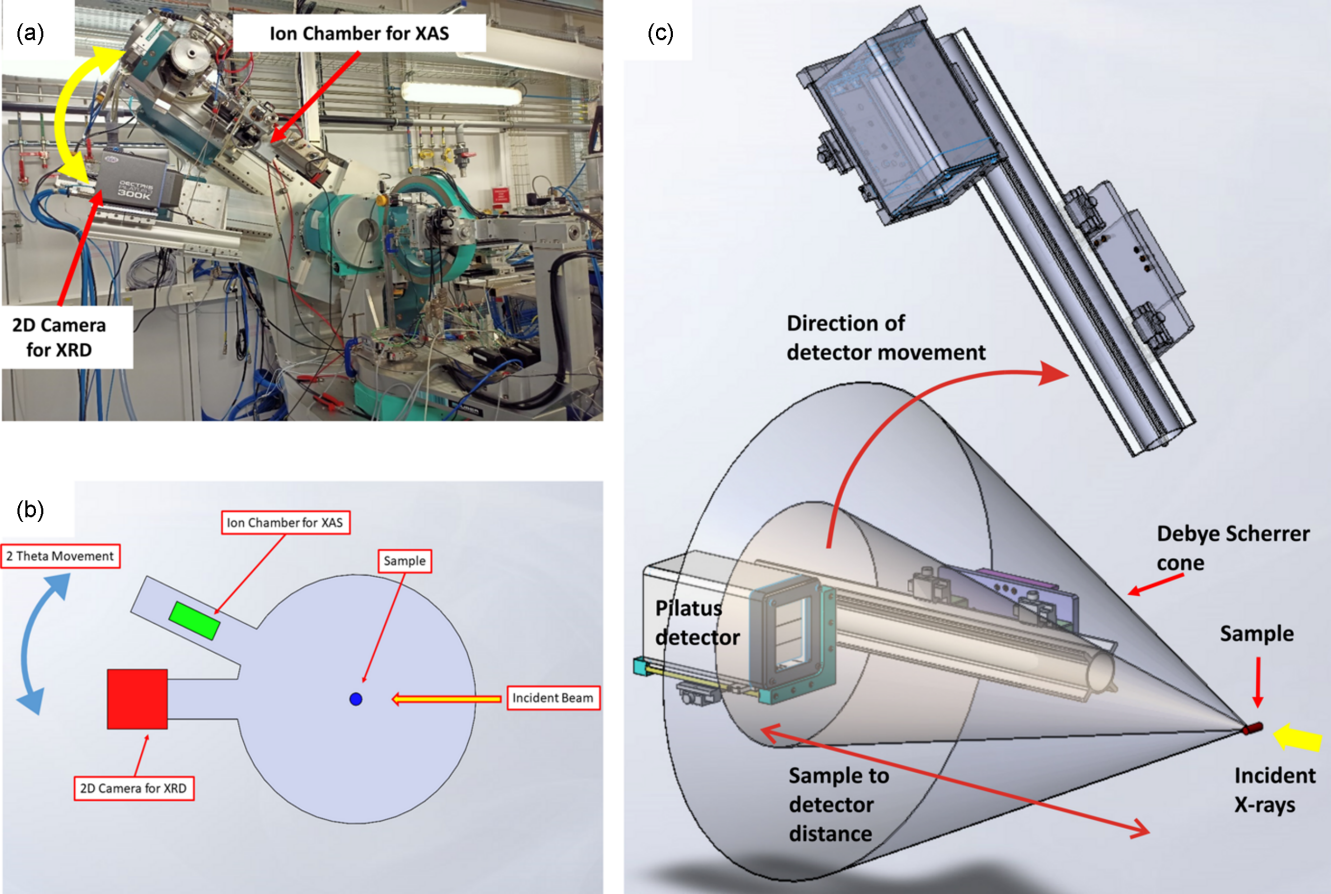
Experimental arrangement for the measurement of anomalous XRPD using a scanning flat-panel detector, showing how it is interleaved with transmission XAS. (*a*) The mounting of the flat-panel detector and ion chambers on independent arms on one axis (in the direction of the X-ray beam) of a six-circle Huber diffractometer. (*b*) and (*c*) Schematic representations indicating the direction and range of movement of the flat-panel (Pilatus) detector.

Fig. 1 ▸ shows the overall experimental arrangement (Thompson *et al.*, 2015 ▸; Thompson *et al.*, 2019 ▸) used on the XMaS beamline to achieve the AXRPD measurements interleaved with XAS measurements made in transmission. The beamline is centred upon an 11-axis Huber diffractometer, which makes many aspects of sample positioning and detector arrangement and displacement both easy to achieve and very precise. Crucially, both the flat-panel detector (Pilatus3 R 300K; Dectris, Switzerland; https://dectriswebsite-live-eac9c2f266324cfaa2c-57347c9.divio-media.com/filer_public/2f/5c/2f5cb8f8-32b4-4de1-962e-10cd808b98b7/technical_specifications_pilatus3_r_300kw_v3.pdf) used for the detection of X-ray scattering and the ion chamber required for the measurement of XAS in trans­mission [Oken ionization chamber (Ohyo Koken Kogyo Co Ltd, Japan), filled with an N_2_/Ar mix] can be positioned alternately at the desired value of 2θ and on-axis to permit easy interleaving of the two measurements. Furthermore, the 11-axis mounting system permits the sample to be precisely positioned and maintained at the centre of rotation on the (horizontal) axis normal to the axis of the X-ray beam and X-ray detection.

The beamline can take a full 50 mm wide fan in the horizontal plane, some 23.6 m away from the 0.86 T short dipole within the ESRF storage ring (Thompson *et al.*, 2015 ▸; Thompson *et al.*, 2019 ▸). The first optical element of the beamline is a liquid-N_2_-cooled Si〈111〉 monochromator, followed by a 1.4 m long chromium toroidal mirror, at 2.5 mrad angle of incidence to the beam, to focus the beam to a spot size of roughly 100 µm^2^ at the sample position. Before the monochromator there is a set of primary slits, and these were used to set the vertical divergence of the beam. After the monochromator and before the focusing toroidal mirror, there is a set of secondary slits that can be used to change the horizontal divergence of the bending magnet fan before the focusing mirror. As there is no pre-mirror, the monochromator is exposed to the full vertical divergence of the source. Therefore, these optics are not optimized for very high energy and wavevector resolution when coupled with a toroidal mirror. The resolution, however, can be enhanced by the use of slits both before and after the monochromator, at the expense of flux transmitted to the sample. For both the AXRPD and XAS measurements, a primary vertical slit of 0.5 mm was used, and the full 50 mm fan was taken onto the liquid-N_2_-cooled monochromator. For XRPD, the horizontal fan of bending magnet radiation was reduced to 10 mm before the toroidal mirror, using the secondary slits, but for the XAS measurements it was opened up to 50 mm to increase the flux, as the increased horizontal beam divergence that results is not critical for these measurements.

The double-crystal monochromator used on BM28 is an ‘in-house’ design, and the second crystal is motorized using three Huber Diffraktionstechnik GmbH high side load actuators with a step resolution of 50 nm. These actuators drive the second crystal using a typical three-point kinematic mount, allowing the distance between the crystals, as well as the pitch and roll, to be easily controlled. Due to the simple optics of the single toroid, coupled with the fixed offset double-crystal monochromator, the energy can be rapidly switched between the copper *K* edge (9 keV) and the energy used for the off-resonant XRD measurement (17.5 keV) (Bikondoa *et al.*, 2019 ▸).

To change the horizontal divergence between the XRPD and XAS measurements, the horizontal slit, after the monochromator and before the toroidal mirror, was moved to avoid altering the heat load experienced by the monochromator. To align the second monochromator crystal, whilst changing the energy between 17.5 keV and the copper *K* edge energy, a single pixel (172 × 172 µm) from the Pilatus camera on the 2θ arm was used as a counter in the attenuated main beam. This permitted alignment of the height, roll and pitch of the second crystal and allowed the beam to be driven back to the same position at the centre of rotation of the diffractometer in a reproducible way. The dimensions of the beam itself at the sample were measured using an ESRF Basler X-ray beam viewer, a Basler camera equipped with a scintillator crystal (Ce-doped YAG: Y_3_Al_5_O_12_:Ce, 250 µm thick), a 1× optics and a 45° mirror, so that the camera chip does not see the direct beam. The pixel size is 3.75 µm and the exposure time can be varied between 10 s and 16 µs.

A motorized tungsten beamstop was placed behind the sample to prevent the 2D camera being exposed to the direct X-ray beam when performing XRPD. This beamstop can be moved when switching between the XRPD and XAS measurements. Both the ion chamber and the 2D Pilatus 300K camera are mounted onto a dual-rail 2θ arm. By moving the 2θ arm, the ion chamber and the camera can be moved in and out of the correct positions (Fig. 1 ▸).

Images of the diffraction rings were saved on the Pilatus 2D camera by moving the 2θ arm from 4° to 54° in 0.25° steps. The images were reduced to 1D diffraction patterns using the *esaProject* software (see supporting information, Section S5). Using this arrangement, complete XRPD patterns, of the sort discussed below, could be collected in *ca* 30 min with the interleaved XANES measurements taking *ca* 20 min. The latter were limited by the monochromator movement rather than any other factor.

The sample environment employed is a modification (Newton *et al.*, 2019 ▸) of that introduced by Chupas *et al.* (2008 ▸), which was originally employed for total X-ray scattering/PDF measurements and therefore permits the detection of scattered X-rays over a very wide angular range as required; it is also capable of achieving sample temperatures of up to 1000°C. Moreover, this sample environment has been extensively verified in terms of the bed dimensions over which it maintains an isothermal heating zone, so that positioning of the sample within the said zone, and the length of the sample bed permitted to be used, are well understood. As has been demonstrated in related systems (Newton *et al.*, 2019 ▸), the presence of axial and radial thermal gradients existing in the absence of any reactive chemistry can lead to considerable over- or underestimates of levels of zeolite dehydration and speciation of the copper and thus of observed reactivity, such that, depending on the sampling location within the bed, completely erroneous views of the progression and kinetics of the chemistry at work will be obtained.

The sample itself is mounted into this arrangement along the centre of rotation and on the horizontal axis normal to the X-ray beam. It is presented as a packed (no more than 5 mm long) bed, secured by small quartz wool plugs, within a quartz capillary of 0.5 mm inner diameter and 0.01 mm wall thickness. These capillaries provide sufficient transmittance for both off-resonance and near-resonance energies, resulting in favourable diffraction data resolution, line profile and signal-to-noise ratio (Fig. S1). Ordinarily within this arrangement, the temperature of the sample would be measured using a thermocouple directly inserted into the exit of the bed. However, the very thin capillary required for the XRPD does not easily permit this. Therefore, the system once mounted must be calibrated for temperature. To achieve this, we used the Si standard measured as described above. The position of the Si(111) Bragg diffraction peak was then measured after power was applied and the experiment allowed to stabilize. This process was repeated over the range of applied voltages and the XRPD images then used to derive a temperature versus power calibration curve, based upon the known coefficient of thermal expansion of silicon, which is given as supporting information (Fig. S2).

### 2.3. Characterization of the setup for XRPD using a silicon standard

**Figure 2:**
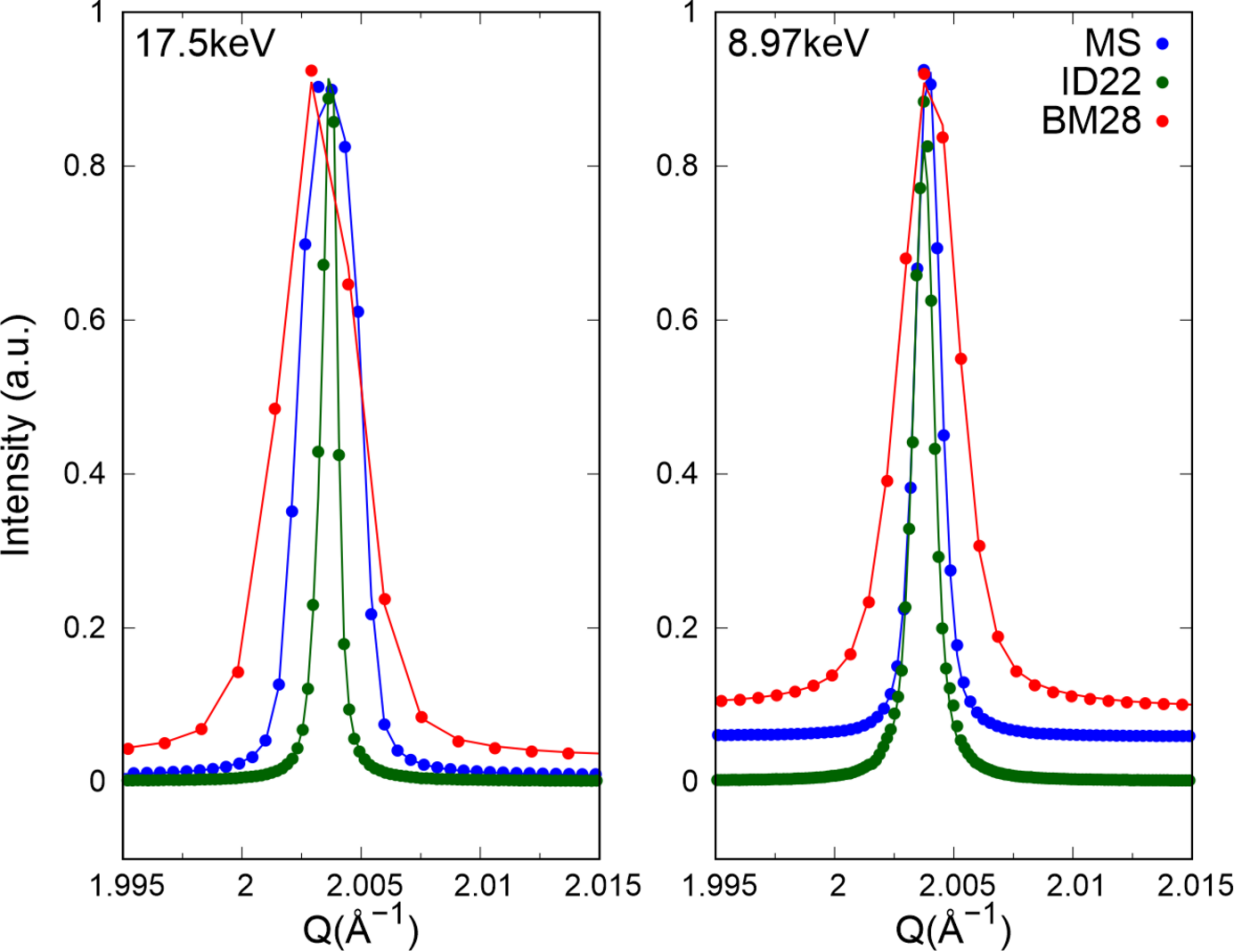
Comparison of the Si(111) reflection obtained from an Si NIST640c standard on beamlines BM28 (XMaS) at the ESRF (red), MS-X04SA at the Swiss Light Source (blue) and ID22 at the ESRF (green). Both MS-X04SA and ID22 are undulator-based beamlines that use Si(111) channel-cut monochromators, whereas the post-ESRF-upgrade XMaS is founded upon a 0.86 T ‘short bend’ and uses a cryogenically cooled Si(111) double-crystal monochromator. In the case of the MS-X04SA beamline a MYTHEN II detector was used. On ID22 a scanning multicrystal analyser stage was used, whereas the results from XMaS were achieved through scanning a 2D detector through the required angular range. Measurements on ID22 and XMaS were also made after the recent ESRF upgrade.

The baseline performance of the beamline and detection system used in the XRPD experiment was initially evaluated using a powder standard (Si NIST640c). Measurements were conducted by scanning the flat-panel detector, as previously described, at three different X-ray energies: 17.5 keV for conventional diffraction and 8.94 and 8.97 keV for anomalous (resonant) powder diffraction near the Cu *K* edge. The resulting diffraction data are presented in Fig. 2 ▸ and compared with reference measurements obtained from two high-precision XRPD beamlines: ID22 at the ESRF (Fitch *et al.*, 2023 ▸) and MS-X04SA at the Swiss Light Source (Gozzo *et al.*, 2010 ▸; Willmott *et al.*, 2013 ▸).

Instrumental profiles were accurately modelled using a pseudo-Voigt function and the simple axial model via the Pawley refinement as implemented in the program *TOPAS Academic* (Coelho, 2018 ▸). Pattern matching against the Si standard [unit-cell parameter *a* = 5.431195 (9) Å] confirmed that the actual X-ray energies deviated by less than 1 eV from the target values on BM28 in all cases. Table 1 ▸ summarizes the FWHM values extracted from the diffraction data.

The BM28 (XMaS) data exhibit noticeably broader diffraction peaks than those measured on ID22 and MS-X04SA. The following factors contribute to the larger FWHM observed on BM28.

(i) Optical arrangement and detector configuration. BM28 utilizes a scanning 2D imaging detector that is moved through the required angular range. This setup, while versatile for *operando* measurements and interleaving with XAS, is not specifically optimized for high-resolution XRPD. In contrast, ID22 and MS-X04SA are dedicated powder diffraction instruments with optimized optical geometries to minimize instrumental broadening.

(ii) Pixel size and angular resolution. The finite pixel size of the flat detector, combined with the relatively long sample-to-detector distance required to maintain angular resolution, contributes to an increased instrumental broadening. ID22 and MS-X04SA employ detectors and optics designed to reduce such effects, resulting in sharper peak profiles.

(iii) Contribution from capillary and sample environment. The capillary dimensions and the sample mounting, although kept as thin as possible to optimize diffraction quality, introduce additional broadening in the BM28 setup. The special­ized setups on ID22 and MS-X04SA benefit from optimized sample environments that minimize these contributions.

(iv) Wavelength effects. Minor differences in the effective X-ray wavelength used in the experiments also influence the FWHM values. The slightly larger FWHM observed at the Cu *K* edge on BM28 (0.035° versus 0.024° at 17.5 keV) reflects the sensitivity of the instrument’s resolution to the X-ray energy.

In summary, the broader FWHM values on BM28 are a result of the experimental configuration, which prioritizes the ability to interleave XRPD with XAS measurements over achieving the resolution offered by beamlines like ID22 or MS-X04SA. These factors explain the approximately 1.5–2 times larger FWHM compared with MS-X04SA and 3–8 times larger FWHM compared with ID22, values that are within expectations for a 2D scanning diffraction setup.

## 3. Application of the method, interleaved with Cu *K* edge XANES, to the aerobic activation of Cu/MAZ

**Figure 3:**
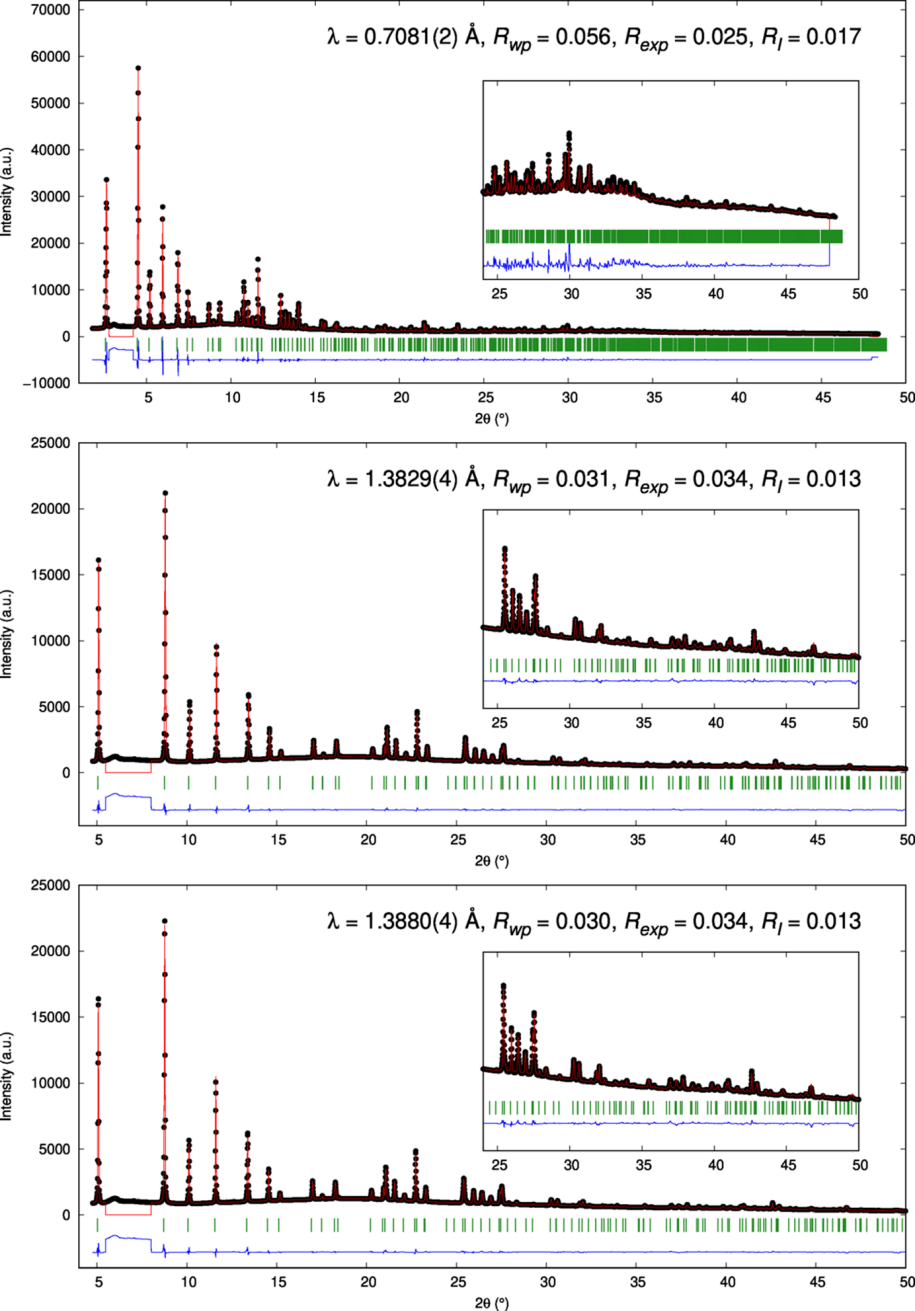
Rietveld refinement profiles (solid symbols) for Cu-MAZ zeolite during activation under flowing O_2_ at 275°C on XMaS and restored from a sequence of images collected using a scanned 2D imaging detector for the three X-ray wavelengths used in this study: λ = 0.7081 Å (17.5 keV), λ = 1.3829 Å (8.97 keV) and λ = 1.3880 Å (8.94 keV). Fits to the data derived in *TOPAS* are given in black.

Fig. 3 ▸ shows AXRPD data (solid circles) and refined models (solid lines) derived on BM28 for a Cu-MAZ sample during its thermal activation under flowing oxygen at 275°C. The data were obtained at one off-resonance energy (17.5 keV) and two on-resonance energies (8.94 and 8.97 keV), as indicated. Fits to the data, derived from analysis using *TOPAS* (Coelho, 2018 ▸), are also given.

**Figure 4:**
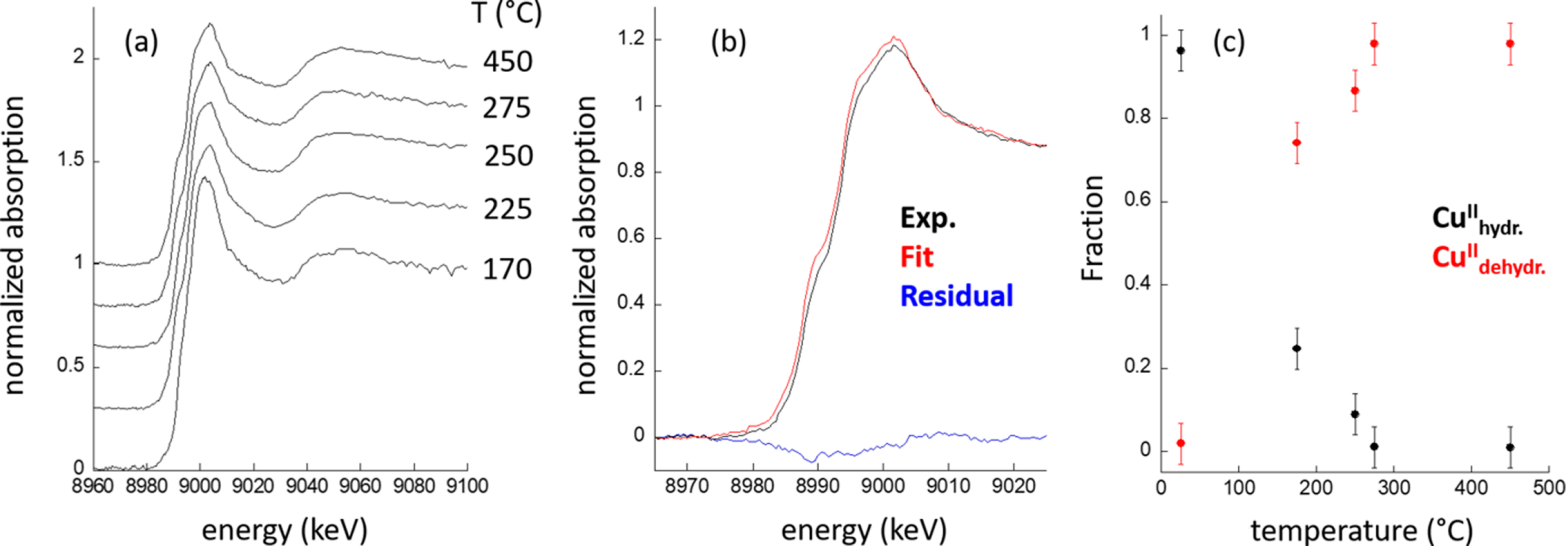
(*a*) Normalized Cu *K* edge XANES collected at each sample temperature (as indicated) for which AXRPD data were subsequently collected from the Cu–MAZ system experiencing an O_2_ flow. (*b*) A comparison (250°C) of the results of LCA analysis and residuals. (*c*) Partitioning of Cu^II^ speciation between hydrated (black) and dehydrated (red) states as a function of temperature as derived from LCA analysis of the Cu *K* edge XANES.

Fig. 4 ▸ shows (*a*) normalized Cu *K* edge XANES collected in between each XRPD measurement at each temperature of investigation, also obtained during the thermal and aerobic activation of Cu-MAZ; (*b*) an example (250°C) of linear combination analysis (LCA) using the *PrestoPronto* package (Figueroa & Prestipino, 2016 ▸) with the residuals (in blue) left by this analysis; and (*c*) the fractions of hydrated (Cu^II^_hydr._) and dehydrated (Cu^II^_dehydr._) copper species present in Cu-MAZ at each temperature that result from the LCA.

Importantly, at each step in the experiment, the XAS shows no evidence of any significant formation of Cu^I^ and the system can, at all points, be well described in terms of Cu^II^ species partitioned between hydrated and dehydrated states. This is an aspect of the speciation that cannot be easily deduced from the XRPD, yet is important in the activation process for Cu–zeolites in respect of the conversion of methane to methanol. Understanding the oxidation state and local coordination environment of the copper species is essential for optimizing the catalytic activity and selectivity of the material. It further shows, in line with, for instance, thermogravimetric analysis of Cu–MAZ (Wieser *et al.*, 2023 ▸), that the system is essentially dehydrated (in terms of the copper) at temperatures of less than 300°C. This correspondence indicates that the calibration of the apparatus (see above and supporting information) in terms of temperature is valid to a good degree.

**Figure 5:**
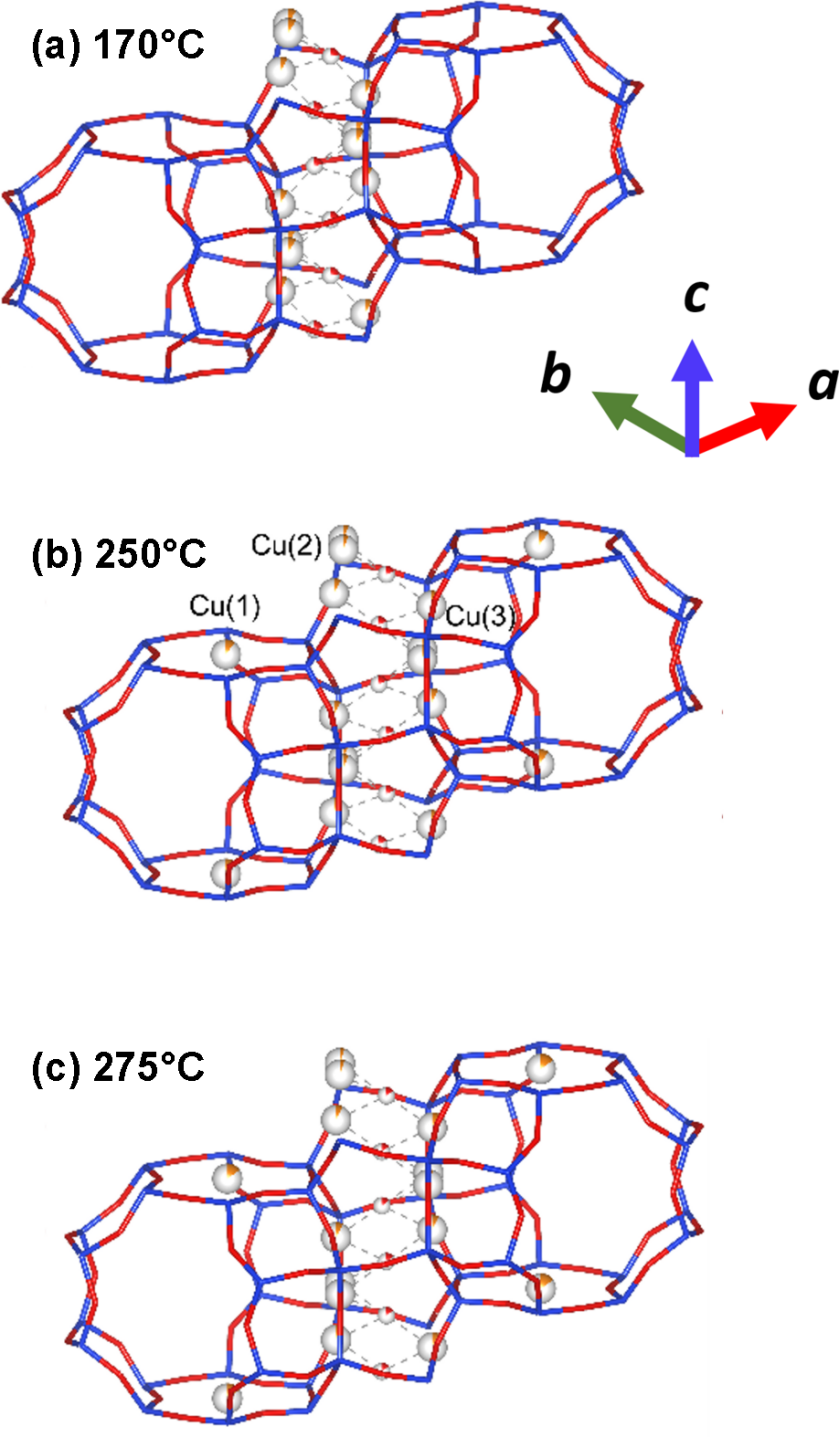
(*a*)–(*c*). Representations of the copper positions within the Cu-MAZ structure as a function of temperature (as indicated) during aerobic activation, as derived from the refinements of the AXRPD data given in Fig. 3. The corresponding initial Fourier maps, for both conventional and anomalous approaches, are given in the supporting information (Fig. S7).

Fig. 5 ▸ then summarizes the results of the analysis of the AXRPD data derived from both standard (monochromatic, 17.5 keV) and anomalous (8.94 and 8.97 keV) approaches to analysis with regard to the disposition and structural partitioning of the Cu^II^ species within Cu-MAZ at three temperatures.

As a general conclusion we note that the distributions of copper positions at 250 and 275°C resemble those derived for the high-*T* activation at 450°C (Knorpp *et al.*, 2021 ▸) and therefore do not, of themselves, explain the significant differences in reactivity and productivities observed between these two reaction temperatures (Wieser *et al.*, 2023 ▸).

However, the results do show that the occupation of the site centred in the six-membered rings of the gmelenite cages arises as a result of the applied treatment and is not native to the synthesis of the materials. At 170°C, the data contain no evidence for the occupation of the six-membered ring site by copper. The electron densities assigned to copper in the six-membered ring appear only above 250°C. As the XRPD shows only two types of Cu – active paired monomers in the eight-membered rings and inactive in the six-membered rings, unless the latter is derived from Cu that is initially disordered and therefore invisible to XRPD – this means that occupation of the six-membered rings must come at the expense of active paired monomers. This in turn implies that, at some point between 170 and 250°C, be it as a result of progressive dehydration, increased temperature or both (as they are naturally correlated), some fraction of the copper migrates from the eight- to the six-membered rings.

We may speculate that, whilst the resulting reactivity/productivity for selective oxidation of methane to methanol in large part results from dehydration of the copper atoms and of the zeolite structure in general, this also leads to a loss of a proportion of the potentially active paired monomers in the eight-membered ring sites and to deactivation, as has been previously observed (Knorpp *et al.*, 2021 ▸). This observation also means that the previously reported loading dependence of yields (Knorpp *et al.*, 2018 ▸; Knorpp *et al.*, 2019 ▸) requires reinterpretation as being the result of the activation treatment, rather than as being an inherent tendency of the Cu to occupy the six-membered ring sites first and only at higher loadings entering the eight-membered ring sites. Nonetheless, the data and analysis given above demonstrate that the methods of data collection that we have proposed and which are the subject of this article can provide a very effective means of achieving *operando* crystallographic data and insights, whilst complemented by a capacity to interleave these measurements with transmission XAS.

## 4. Discussion

The advantages of the method used to acquire the AXRPD are an arbitrarily large 2θ range, a large dynamic range, high sensitivity, good resolution, a high signal-to-noise ratio, low relative statistical fluctuation, an ability to correct for camera defects such as hot, locked or saturated pixels, and an ability to interleave the acquisition with other techniques. These and other advantages of the image merging method used here are described in more detail by Dowsett *et al.* (2020 ▸).

The approach to anomalous (resonant) X-ray powder diffraction that we have described here, and specifically its application to *in situ* studies, has widespread potential application. This method can be seen as existing between the implementation of static flat-panel detectors, 2θ scanning multi-crystal detectors and arc detection systems, which permits simple interleaving with transmission XAS measurements and fuses desirable aspects of the two methods.

However, in terms of the speed of data acquisition in our particular measurement sequence and the ability to achieve full XRPD patterns in a single shot, this approach cannot compete with either static flat-panel systems or arc detectors, simply as a result of the need to scan the detector through the required angular range. Nevertheless, in cases where only a fraction of the angular range requires investigation, *e.g.* to establish the kinetics of phase change, the method permits simple isolation of specific areas within the available *Q* (2θ) range, where the extremely fast frame rates of modern flat-panel detectors can then be brought to bear whilst retaining good angular resolution within the field of view. Furthermore, as such detectors can be gated and very precise timing achieved, the method that we have outlined could find application in high-dynamic structural studies of reversible systems (pump–probe experimentation) and provide textural information.

All that said, in this first demonstration we have not attempted to optimize the rates of data acquisition for either XRPD or XANES, and in both cases faster measurement times seem readily obtainable according to the materials under study and the objectives of the experiment. This is especially the case for XAS, with the replacement of the monochromator used in this case with a new design that allows the required energy ranges to be scanned much more quickly.

A further observation, in regard of possible refinements of the approach, is that, in this first implementation, various path lengths (for instance between the sample and the detectors used for both XRPD and XAS) are somewhat long. For the XRPD component, as a result of the finite pixel size of the detectors, this has to be the case to retain the required angular resolution. Such path lengths significantly reduce the efficiency of detection, especially in the case of resonant X-ray measurements. For instance, an air path length at ambient temperature and a pressure of 1 atm result in the loss of slightly over 50% of the scattered or transmitted X-ray flux at 9 keV (Cu *K* edge) (Henke *et al.*, 1993 ▸), while these losses increase to 90% at the Fe *K* edge and essentially 100% at the Ti *K* edge. However, if, in front of both detectors, suitable flight tubes were installed, either maintained under vacuum or helium purged, this limitation could be overcome, efficient detection of both XRPD and XAS restored, and the method extended toward the tender X-ray regime, albeit with a reduced *Q* (2θ) range for any anomalous investigation. Alternatively, the implementation of a flat-panel detector having significantly smaller pixels than the Pilatus 300K (*e.g.* Maxipix) could also be used to reduce these path lengths significantly whilst not inducing any reduction in the resolution of the XRPD experiment.

In those cases where transmission XAS is no longer viable and fluorescence detection is required, the flexibility of the setup means that this too could easily be accommodated. So too could, at least in principle and in either a standard or a backscattering geometry, high-energy-resolution fluorescence detection XANES, or other spectroscopic variants of emission spectroscopy.

## 5. Conclusions

We have used and extended a previously described approach (Dowsett *et al.*, 2020 ▸) for the collection of *in situ* anomalous (resonant) powder diffraction and shown how XAS measurements can easily be interleaved with it. The resulting crystallographic-quality XRPD data can be used to determine the atomic positions of reactive copper atoms in a Cu–MAZ zeolite crystal structure during variable-temperature and aerobic activation. These findings advance our mechanistic understanding of selective methane oxidation to methanol over Cu–zeolites. The observed migration of Cu species from active paired monomers in eight-membered rings to isolated sites in six-membered rings upon thermal activation suggests that maintaining copper in its active environment is critical for maximizing catalytic performance. This insight emphasizes that both dehydration dynamics and copper siting must be carefully balanced during catalyst activation to preserve high methanol yields, as was also shown by Wieser *et al.* (2024 ▸).

XAS reveals aspects of the chemical speciation of the copper (most specifically the partitioning of Cu^II^ in both hydrated and dehydrated states) at each step, and these can be quantified.

The experimental geometry is inherently open, allowing the introduction of various additional elements without compromising the core measurement. For example, fluorescence detectors (*e.g.* silicon drift detectors) can be positioned without significant obstruction to the XRPD detector path, as can detectors required for transmission-based measurements [*e.g.* XAS or small-angle X-ray scattering (SAXS)]. The scanning mechanism used for AXRPD requires only that the detector can be scanned with sufficient precision in one dimension over the required angular range. This setup does not preclude the placement of other detectors in transmission or backscattering geometry or at 90° to the incident X-ray beam, typical for techniques such as XAS, X-ray emission spectroscopy (using either Johann or von Hamos geometries) or SAXS. Furthermore, the fundamental requirement of the measurement – precise scanning of a suitable detector across the desired angular range – should be readily achievable on many existing, often spectroscopy-based, beamlines, provided that the requirements for pre-sample beam conditioning and sample-to-detector distances (with respect to resolution) can be met. This makes the method broadly applicable across a range of facilities.

Beyond the demonstration of the method, which can be seen to have a number of distinct advantages, we suggest that this approach to AXRPD could have widespread application in many fields of materials research wherein the precise and *operando* understanding of structure and speciation is highly sought after. This combined AXRPD–XAS methodology can be readily adapted to investigate a wide range of crystallographic systems beyond zeolites, including complex oxides, metal–organic frameworks, battery electrode materials and heterogeneous catalysts – anywhere where simultaneous insight into complex atomic structure and elemental speciation under working conditions is critical and where the intrinsic nature of the materials under study permits the method to be applied.

## 6. Related literature

The following additional references are cited in the supporting information: Cromer & Liberman (1981 ▸); Järvinen (1993 ▸); Stephens (1999 ▸).

## Supplementary Material

Additional experimental details. DOI: 10.1107/S1600576725007022/vb5089sup1.pdf

## Figures and Tables

**Table 1:** FWHM values extracted from diffraction data for beamlines BM28, ID22 and MS-X04SA

Beamline	X-ray energy	FWHM (° 2θ)
BM28 (XMaS, ESRF)	17.5 keV	0.024
BM28 (XMaS, ESRF)	Cu *K* edge (8.94 and 8.97 keV)	0.035
ID22 (ESRF)	17.5 keV	0.003
ID22 (ESRF)	Cu *K* edge	0.012
MS-X04SA (SLS)	17.5 keV	0.016
MS-X04SA (SLS)	Cu *K* edge	0.018

The FWHM values correspond to the reflection observed from the Si(111) peak, as depicted in Fig. 2.

## Data Availability

Data supporting the findings of this study are available within the article and the supporting information. All raw data generated during the study can be requested from P. Rzepka and M. A. Newton.
