# Supplementary material for: Operando anomalous X-ray powder diffraction interleaved with X-ray absorption spectroscopy using a scanning 2D imaging detector on the XMaS beamline: design, implementation and performance
Source: J Appl Crystallogr. 2025 Sep 18;58(Pt 5):1778–88. doi: 10.1107/S1600576725007022 (PMC12502866; doi:10.1107/S1600576725007022)
Supplement: Supplementary file 1 [file j-58-01778-sup1.pdf]

JOURNAL OF APPLIED CRYSTALLOGRAPHY

Volume 58 (2025)

Supporting information for article:

Operando anomalous X-ray powder diffraction interleaved with X-ray absorption spectroscopy using a scanning 2D imaging detector on the XMaS beamline: design, implementation and performance

Dariusz Wardecki, Paul B. J. Thompson, Kinga Mlekodaj, Mark G. Dowsett, Mieke Adriaens, Amy. J. Knorpp, Catherine Dejoie, Kinga Góra-Marek, Jeroen A. van Bokhoven, Mark A. Newton and Przemyslaw Rzepka

1. Sample preparation and characterisation
2. Absorption versus energy
3. Temperature calibration
4. Experimental setup
5. Data processing, workflow and the synthesis of AXRPD data from the acquisition.
6. XRPD data, fitting, and outputs
7. Fourier maps derived from conventional (17.5 KeV) and Anomalous (8.94 and 8.97 keV) diffraction measurements.
8. The capillary displacement parameters
9. Structural Data for Cu-MAZ under flowing O2 at 170°C
10. Structural Data for Cu-MAZ under flowing O2 at 250°C
11. Structural Data for Cu-MAZ under flowing O2 at 275°C
12. Supplementary references

S1. Sample preparation/characterisation

The overall synthesis and characterisation of the materials here is reported in Knorpp et al., 2018; Knorpp et al., 2019 and Knorpp et al., 2021 of the main paper but is briefly summarised thus:

Zeolite omega was synthesized in its sodium form using an in-house built rotating oven. The synthesis time was 20 days at 110 °C. Post synthesis, the sample was calcined in air at 550 °C for eight hours (ramp rate of 1 K/min).[40] The material was then ion-exchanged with 2 M NH_4NO_3 solution (RTP, 24 hours). The sample was then ion-exchanged with 0.0025 M $Cu(NO_3)_2$ solution (RTP, 24 hours) a total of three times. After each ion exchange the sample was washed with water and ethanol.

Copper, aluminium, and silicon content was determined using a SpectraAA 220FS spectrometer. 10 mg of zeolite was digested in 2 ml of concentrated hydrofluoric acid and 3 ml of concentrated nitric acid overnight and then diluted to 50 ml with deionized water. Calibration curves were prepared from standard solutions. The final Cu/MAZ material exhibits a Si/Al of 4.3 and a Cu loading of 4.4 wt %.

S2. Absorption versus X-ray energy

Capillary sample absorption was estimated based on calculated f' and f'' values using APS online absorption calculator the Cromer & Liberman algorithm [S1] and orbital cross-section tables. Figure S1 illustrates the sufficient transmittance through the capillary at the Cu K-edge and at 17.5 keV.

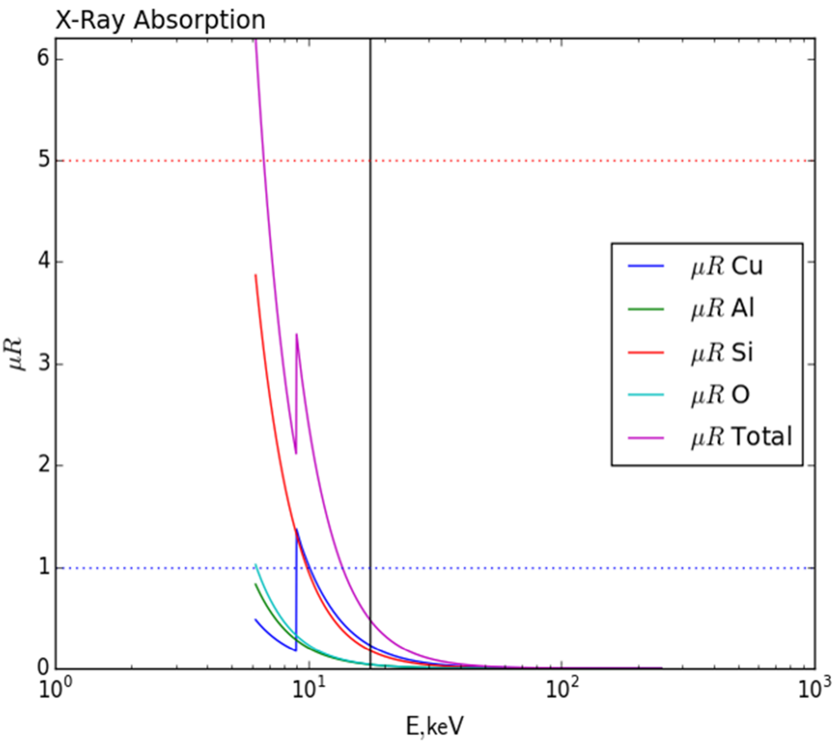

Figure S1 X-ray mass attenuation coefficients mμ for each input element and for the $Cu_2Al_8Si_{28}O_{72}$ composition as a function of X-ray energy. The capillary radius R is 0.5mm and estimated packing fraction 0.6. The blue dotted line indicates a muR value of 1. In a Debye-Scherrer geometry, it is ideal when muR is 1 or below, as sample absorption is minimal and no correction is needed. The red dotted line indicates a muR value of 5. For muR >= 5, measurements are usually not possible in a Debye-Scherrer geometry, as there will be very severe level of absorption and correction is inaccurate. Data from APS online absorption calculator (https://11bm.xray.aps.anl.gov/absorb/).

S3. Calibration of temperature using XRD derived from the Si (NIST standard)

Figure S2. shows the calibration of sample temperature versus applied voltage derived from the Si measured within the reactor setup used for the measurements on Cu-MAZ.

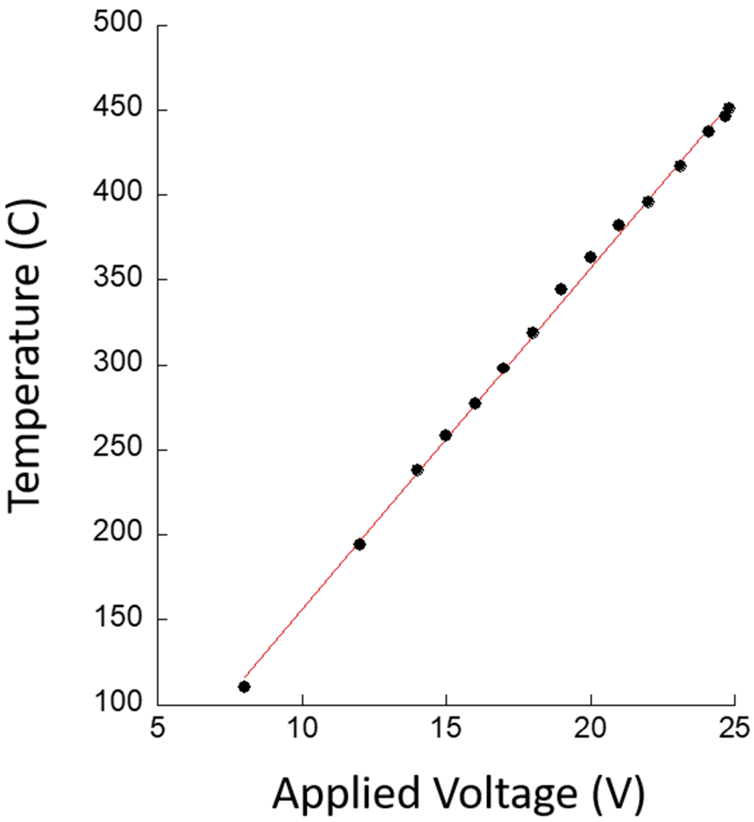

Figure S2 Temperature of the NIST640 Si standard, as measured using the variation in position Si(111) Bragg reflection as a function of the voltage applied to the sample heater system.

S4. Experimental setup

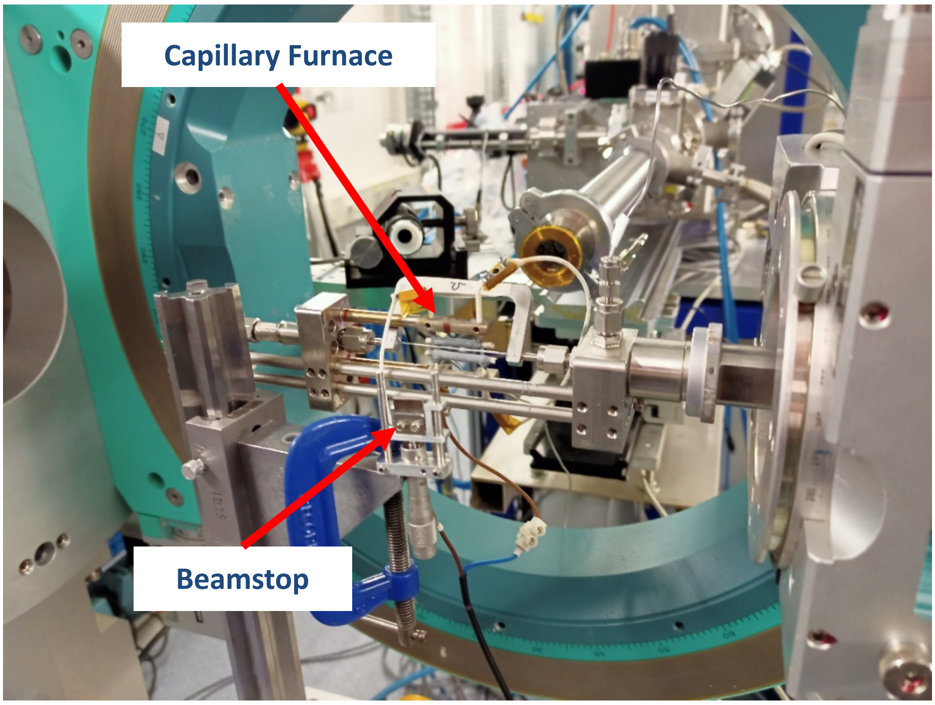

Figure S3 The sample reactor and its mounting at the centre of the axis of rotation along with the positioning of the beam stop.

S5. Data processing, workflow and the synthesis of AXRPD data from the acquisition

This section describes how the sequentially collected images are rendered into AXRPD that can then be submitted to crystallographic analysis.

Figure S4 describes the overall workflow and concepts that lie behind the data processing required to achieve this as implemented by the esaProject (on request from M. Dowsett: markdowsett.esa@gmail.com) software used in this study to transform the sequentially acquired images into a diffractogram.

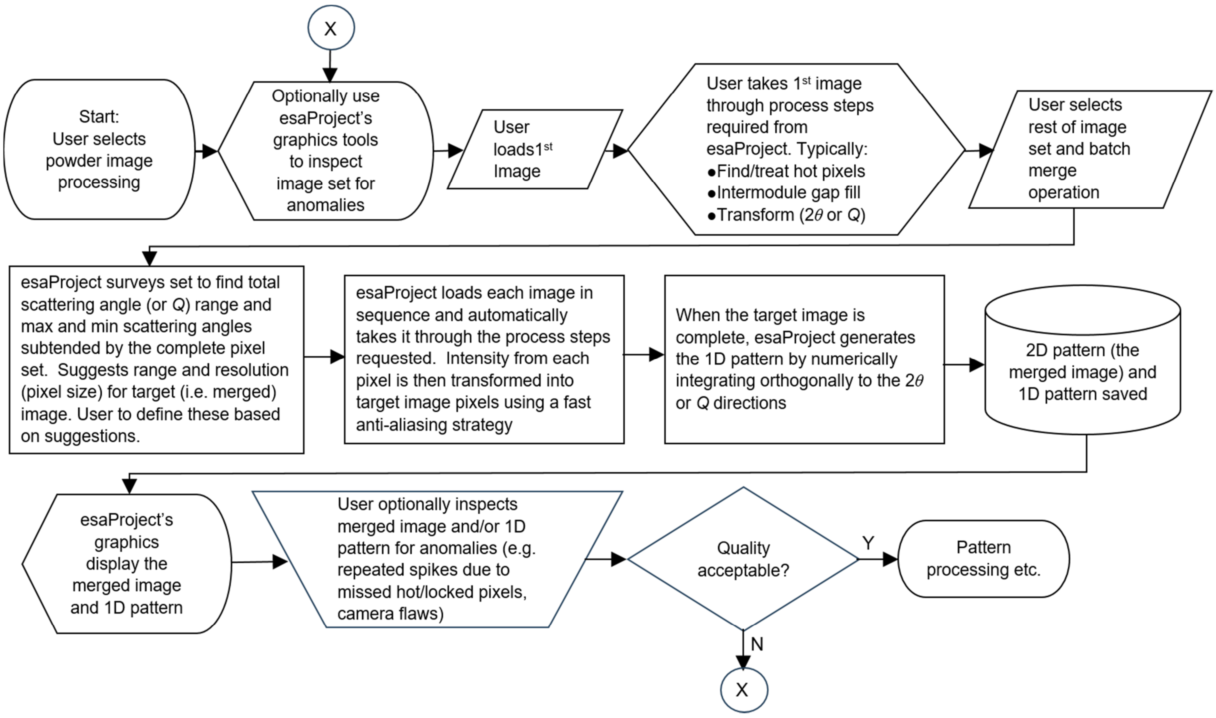

Figure S4 Schematic description of decision-making steps and workflow implemented in esaProject software for the merging of the images sequentially acquired over the angular range permitted by the experimental geometry and sample environment.

The first process step is to quickly examine each image in the set for anomalies. In this case, the Pilatus camera used occasionally outputs pixels locked at a very high intensity (hot pixels) and these need to be treated before merging or they will result in repeated high intensity spikes in the final 1D pattern. esaProject will load and display each image allowing a full zoom down to the single pixel level so that hot pixels may easily be distinguished from small diffraction spots. It is wise to examine the whole set because the flaw can develop after acquisition commences, and the pixels involved may change during the scan.

esaProject has several strategies for finding and removing hot pixels, and the selection of these is camera dependent. esaProject learns the desired image processing sequence from a user-controlled step by step treatment of the first image in the set (the walk through).

Here, the patterns are extracted from the merger of images acquired sequentially on each monochromator step, so even a single hot pixel on the sensor will be very noticeable and produce repeated spikes in the resulting pattern. The Pilatus camera used here is sometimes hot pixel-free and rarely develops more than 2 or 3 hot pixels whilst acquiring an image sequence. These may be present at the start of acquisition, or appear (or disappear) as it proceeds. With repeated use of the same camera months or years apart, we do not observe a fixed pattern of hot pixels so it is not possible to design a simple mask, and the phenomenon does not appear to be associated with camera damage or

extraneous radiation (hot pixels are single, never tracks) but rather the failure of a high intensity pixel to reset immediately.

With the Pilatus3 R 300 K, it is usually sufficient, in the first step in the walk through, to set a threshold a little below the theoretical maximum output (1048573 counts (Technical specifications, Pilatus R 300 K)) and request that more intense pixels are not transformed. The software keeps track of how many pixels are transformed into each column and normalizes the column sum (which is one point in the 1D pattern) to this number (which may be fractional, see below).

Following the survey, and with reference to Figure S3, the first image is loaded and normalized to the beam monitor value in its header or elsewhere. The user selects the hot pixel strategy from the options esaProject presents. If the camera is mounted with the intermodule gaps (Technical specifications, Pilatus R 300 K) orthogonal to the scan direction esaProject needs to fill the gaps by copy-pasting the corresponding strip of pixels from the next image in the set. If this is not done, the 1D pattern will contain negative going spikes whose width will vary with the position in the transformed image. The desired transform is then applied to the first image. The rest of the set to be merged is selected and esaProject finds the maximum and minimum angles (or Q values) subtended by pixels in the complete set as well as the total angular (or Q) range. These are presented to the user who can then define a useful total range and resolution for the merged image. Details of the transforms and anti-aliasing strategy can be found in the esaProject manual (on request from M. Dowsett: markdowsett.esa@gmail.com).

As a final optional step, esaProject can compute the total unnormalized (i.e. integer) intensity of all the pixels used from the image set and restore the total intensity of the 1D pattern to this value. Whilst the 1D pattern will no longer be integer, the relationship between absolute intensity and statistical fluctuation (e.g. for Poisson statistics) is restored by this process which is important for onward processing such as fitting.

esaProject displays the merged image for inspection and saves this image (binary) and the 1D pattern (text). The latter can be loaded into esaProject's pattern and spectrum processing window for comparison with the displayed image.

For around 100 of the 487 × 609 pixel^2 images from the Pilatus, the automatic process described above takes a few seconds on a multicore high-end laptop. It would be possible to transform straight from the image set to the 1D pattern. However, we have found the transformed merged image to be invaluable in identifying features in the 1D pattern due to diffraction spots from large single crystals in heterogeneous materials, spikes due to uncorrected hot pixels, and beam reflection or backscattering. Therefore, we retain it.

S6. XRPD data, Rietveld analysis, and outputs

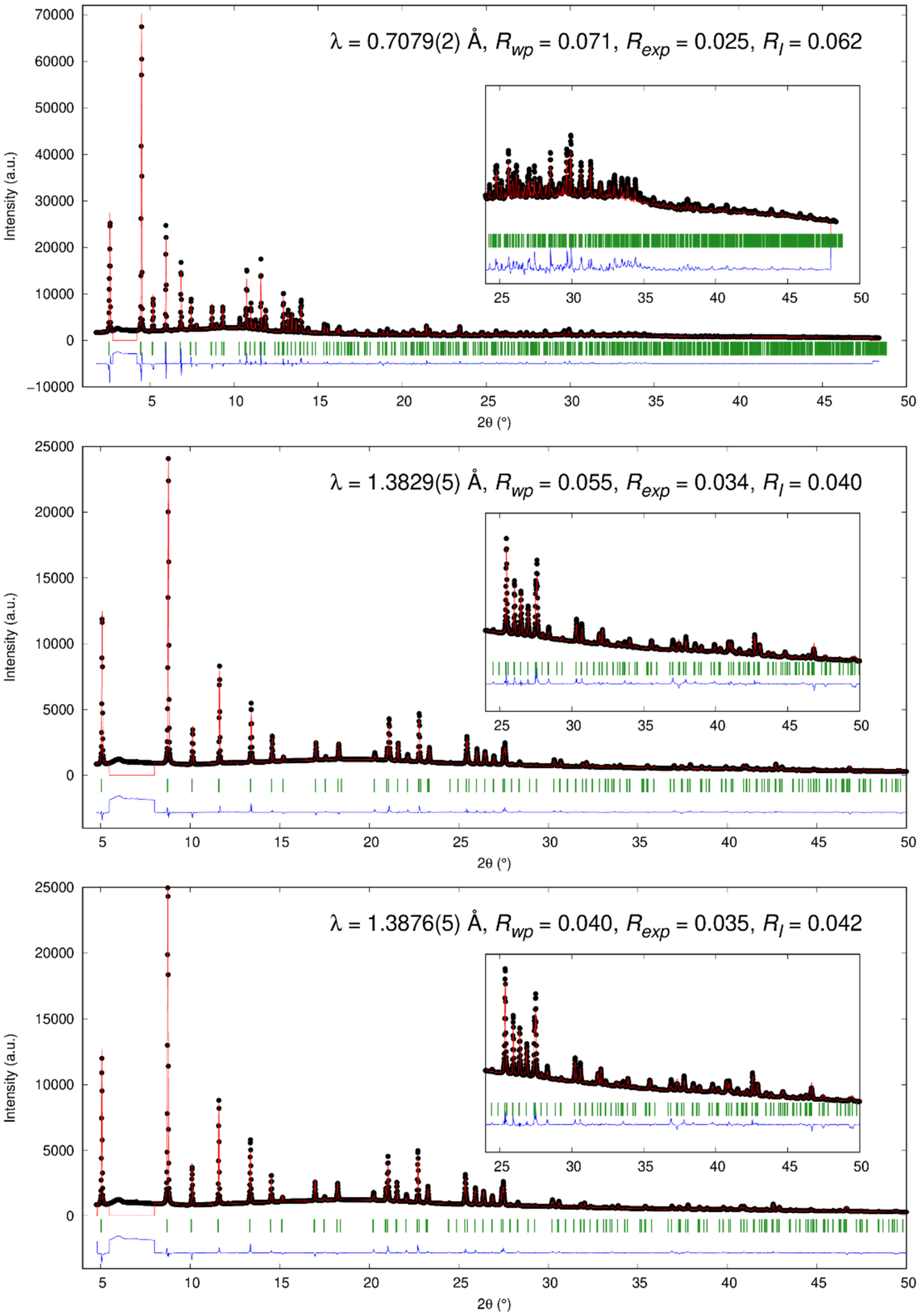

Figure S5 Rietveld refinement profiles (red lines) for Cu-MAZ zeolite data (black dots) collected at the three X-ray wavelengths λ= 0.7079Å (17.5 keV), 1.3829 Å (8.97 keV), 1.3876 Å (8.94 keV). The activation process occurred under flowing O2 at 170°C on XMaS and the profiles were reconstructed from a sequence of images acquired using a scanned 2D-imaging detector.

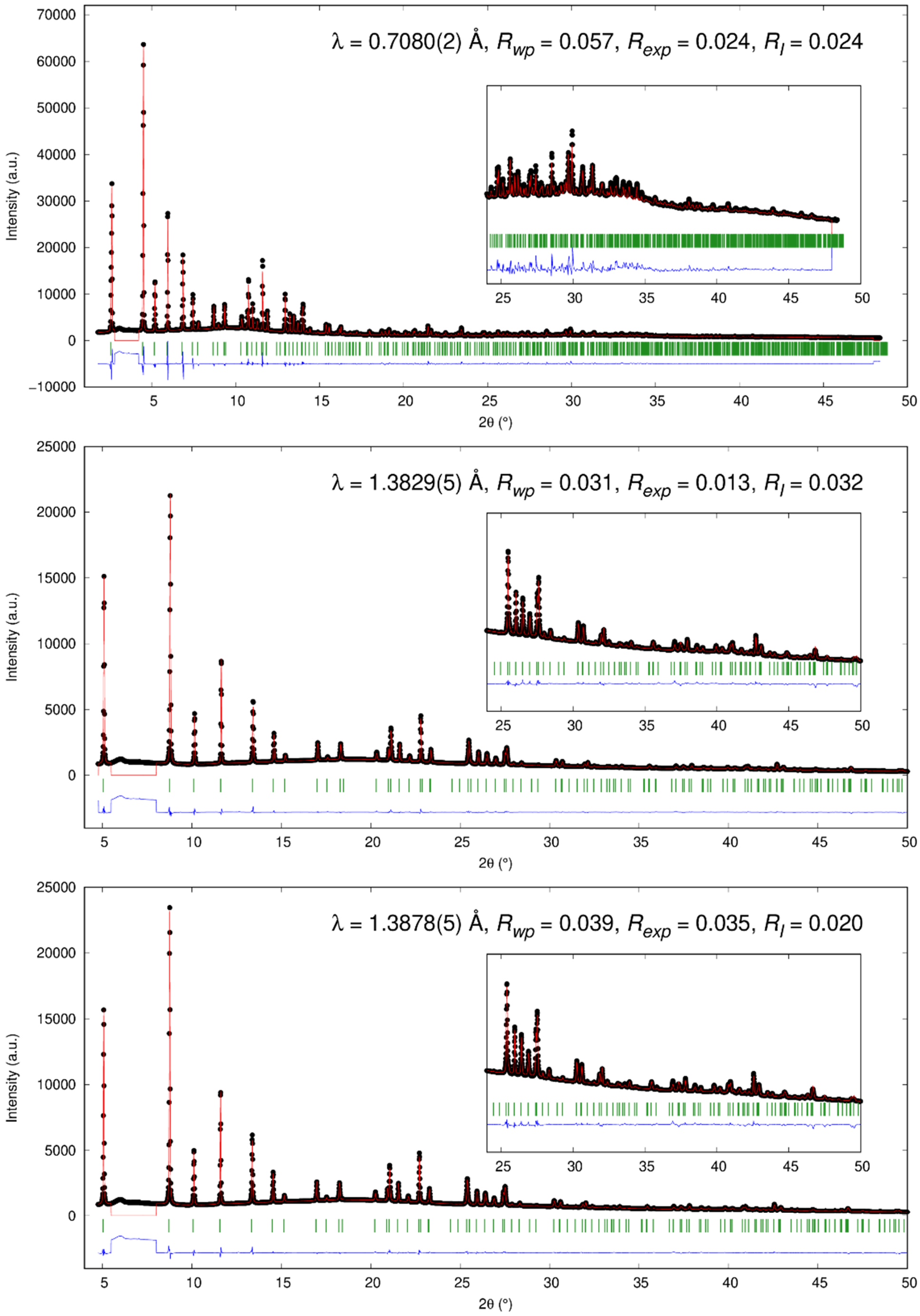

Figure S6 Rietveld refinement profiles (red lines) for Cu-MAZ zeolite data (black dots) collected at the three X-ray wavelengths λ= 0.7080Å (17.5 keV), 1.3829 Å (8.97 keV), 1.3878 Å (8.94 keV). The activation process occurred under flowing O2 at 250°C on XMaS and the profiles were reconstructed from a sequence of images acquired using a scanned 2D-imaging detector.

To accurately model the Bragg peak shapes in our diffraction data, we employed a tailored fitting strategy to account for instrumental and processing effects, particularly at low scattering angles. For measurements collected with a wavelength of λ = 0.7 Å (17.5 keV), the first Bragg reflection, corresponding to the (010) plane, appears at approximately 2.5° 2θ. This peak exhibits significant asymmetry compared to reflections at higher angles. To address this, we modeled its shape and broadening using a dedicated set of parameters, implemented via an IF(...) conditional statement in TOPAS [S2]. The asymmetry was described using the circles_conv function, convoluted with the gauss_fwhm and lor_fwhm functions to capture the peak profile accurately.

For the remaining Bragg reflections at higher 2θ in the 0.7 Å dataset, the same functional forms were applied but with a different parameter set, as these peaks did not exhibit the pronounced asymmetry observed for the (010) reflection. Additionally, anisotropic broadening effects at higher angles were incorporated through a linear combination of sixth-order symmetrized spherical harmonics [S3], introducing four extra free parameters (c20, c40, c60, c66p). Compared to alternative broadening models [S4], this approach provided superior fit quality.

It is noteworthy that the asymmetry observed in the (010) reflection is specific to the data collected at 0.7 Å. In contrast, for measurements performed with a longer wavelength of λ = 1.38 Å (8.9 keV), the (010) peak appears at a higher 2θ angle (around 5°) and does not display significant asymmetry, allowing it to be modeled with a standard set of functions.

```
spherical_harmonics_hkl sh1
sh_order  6 load sh_Cij_prm {
y00   !sh1_c00  1.00000
y20   sh1_c20  -0.14159`_0.00366
y40   sh1_c40  -0.06425`_0.00696
y60   sh1_c60  -0.06492`_0.00801
y66p  sh1_c66p -0.04907`_0.00309}

prm   !pr5    -0.08755
prm    pr6    0.02000` min 0
prm    pr7    0.16650`_0.00577 min 0
prm    pr8    0.01301`_0.00041 min 0
prm    pr9    0.00463`_0.00553_LIMIT_MIN_0 min 0
prm    pr0    0.00715`_0.00035 min 0
prm   !pr1     -0.12632
prm    pr2    -0.00341`_0.25884
prm    pr3    -0.00341`_0.25881
prm    pr4    -0.06619`_0.00175
prm   !pr10    0.01147 min 0
prm   !pr11    0.00444 min 0
prm    pr12   -0.10000`
gauss_fwhm = If(And(H==0,K==1,L==0), pr10, sh1 (pr7*Tan(Th) + sh1 pr8/Cos(Th)));
lor_fwhm = If(And(H==0,K==1,L==0), pr11, sh1 (pr9*Tan(Th) + sh1 pr0/Cos(Th)));
'circles_conv  =  If(And(H==0,K==1,L==0), pr5, sh1 pr2 / Tan(2Th));
'circles_conv  =  If(And(H==0,K==1,L==0), 0.03705, sh1 pr3 / Tan(2Th));
'exp_conv_const = If(And(H==0,K==1,L==0), pr1, pr4);
```

S7. Difference Fourier Maps

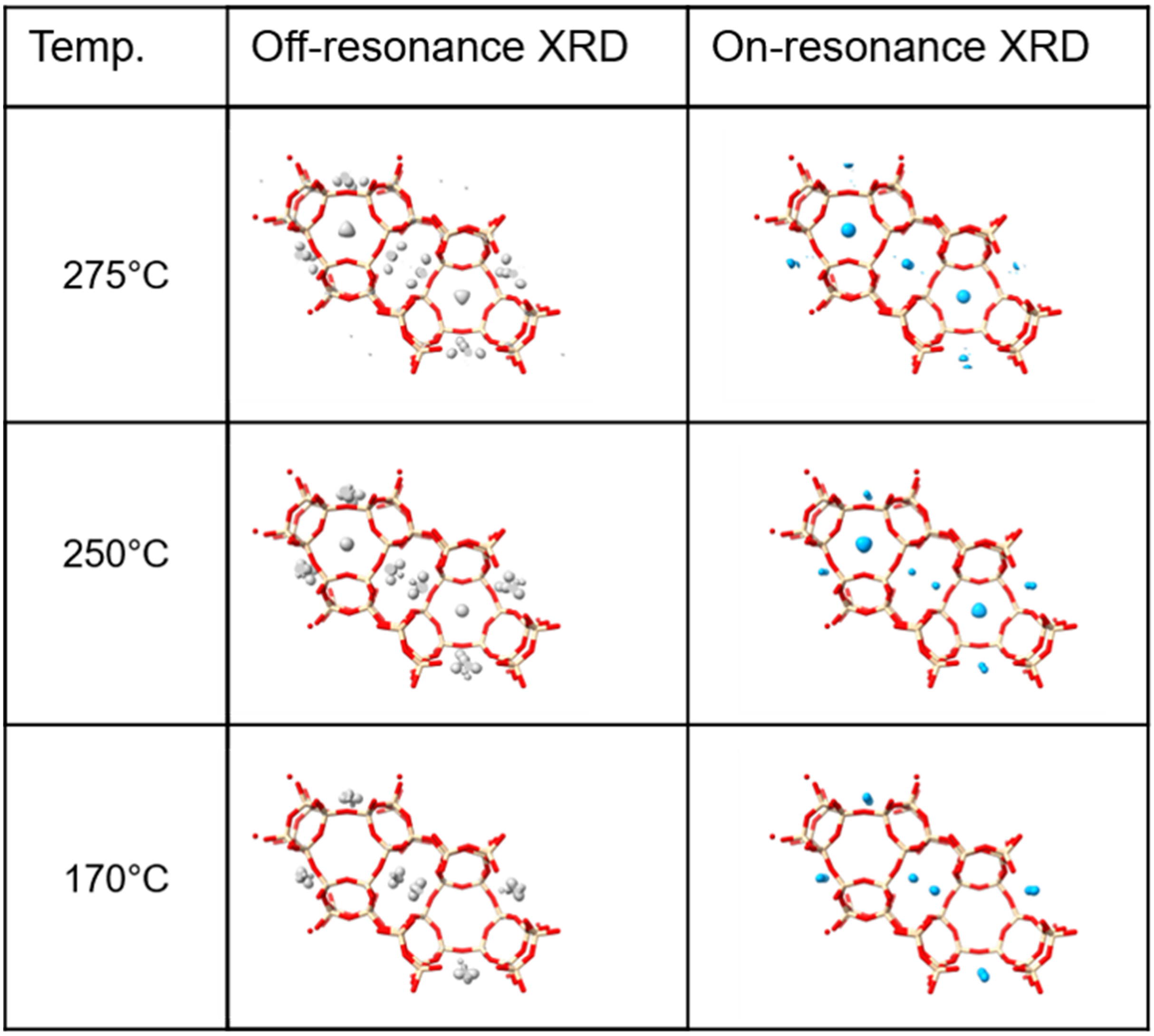

Figure S7 Difference Fourier maps (DMF) derived from conventional (17.5 KeV) and anomalous diffraction data.

Figure S7 depicts the Difference Fourier Maps (DMF) generated by: 1) calculating structure factors (F_{calc}) from the MAZ framework structure model and 2) subtracting the obtained (F_{calc}) from the observed (F_{obs}) measured from Cu-omega at a given temperature. The differential electron densities reveal a significant presence in the 8MR and the peak at 6 MR. The latter disappears at lower temperatures down to 170°C. The densities that vary between off-resonance and on-resonance data were assigned to copper, while others were interpreted as extra-framework oxygen

S8. The Capillary Displacement Parameters

In the course of refinement, we used a constant silicon lattice parameter (a = 5.431195 Å) and refined the common displacement parameters simultaneously for all BM28 datasets. This strategy enabled the simultaneous refinement of both the energy scale and displacement parameters, ensuring that the energy deviations are maintained at less than 1 eV. The table below summarizes the refined parameters along with their uncertainties:

ID	Energy (keV)	Δ Energy	x	Δ x	y	Δ y	Lattice (Å)
218	17.510134	0.001898	0.124629	0.022845	-0.127400	0.001877	5.431195
203	8.941369	0.000922	0.124629	0.022845	-0.127400	0.001877	5.431195
200	8.960568	0.000930	0.124629	0.022845	-0.127400	0.001877	5.431195
MS_175	17.502144	0.000044	0.082122	0.000576	-0.002572	0.000086	5.431195
MS_897	8.972654	0.000225	0.054306	0.006970	0.056691	0.003192	5.431195
MS_898	8.982704	0.000223	0.054306	0.006970	0.056691	0.003192	5.431195
ID22_35	35.000230	0.000034	0	N/A	0.000242	0.000016	5.431195
ID22_897	8.971632	0.000008	0	N/A	0.001391	0.000078	5.431195

These results confirm that our simultaneous refinement procedure effectively corrects for the low-Q shift observed at 17.5 keV and ensures that the energy calibration is robust across all datasets. The energy deviations are indeed maintained at less than 1 eV across all datasets.

S9. Structural Data for Cu-MAZ under flowing O_2 at 170°C

data_ MAZ_170C

_cell_length_a 18.195(6)

_cell_length_b 18.195(6)

_cell_length_c 7.622(2)

_cell_angle_alpha 90

_cell_angle_beta 90

_cell_angle_gamma 120

_cell_volume 2185.3(16)

```
_symmetry_space_group_name_H-M P63/mmc
loop_
    _symmetry_equiv_pos_as_xyz
    'x, y, z '
    '-x, -x+y, z+1/2 '
    '-x, -y, -z '
    '-x, -y, z+1/2 '
    '-x+y, -x, -z+1/2 '
    '-x+y, -x, z '
    '-x+y, y, -z+1/2 '
    '-x+y, y, z '
    '-y, -x, -z+1/2 '
    '-y, -x, z '
    '-y, x-y, -z+1/2 '
    '-y, x-y, z '
    'y, -x+y, -z '
    'y, -x+y, z+1/2 '
    'y, x, -z '
    'y, x, z+1/2 '
    'x-y, -y, -z '
    'x-y, -y, z+1/2 '
    'x-y, x, -z '
    'x-y, x, z+1/2 '
    'x, y, -z+1/2 '
    '-x, -x+y, -z '
    'x, x-y, -z+1/2 '
    'x, x-y, z '
loop_
```

_atom_site_label

_atom_site_type_symbol

_atom_site_symmetry_multiplicity

_atom_site_fract_x

_atom_site_fract_y

_atom_site_fract_z

_atom_site_occupancy

_atom_site_B_iso_or_equiv

Si01 Si 12 0.6684(3) 0.5087(3) 0.25 1 3.375405

Si02 Si 24 0.73822(19) 0.64591(17) 0.5480(3) 1 3.375405

O003 O 12 0.7073(5) 0.6172(5) 0.75 1 4.910439

O004 O 12 0.8406(2) 1.6813(5) 0.5056(9) 1 4.910439

O005 O 24 0.6746(4) 0.5656(3) 0.4349(6) 1 4.910439

O006 O 12 0.7313(5) 0.7313(5) 0.5 1 4.910439

O007 O 6 0.5747(4) 0.4253(4) 0.25 1 4.910439

O008 O 6 0.7437(4) 0.4874(8) 0.25 1 4.910439

Cu1 Cu+2 2 0.6666667 0.3333333 0.25 0.000(5) 5

Cu2 Cu+2 12 0.4537(13) 0.5463(13) 0.504(5) 0.053(2) 5

Cu3 Cu+2 12 0.403(3) 0.497(2) 0.25 0.052(2) 5

O11 O-2 12 0.49314 0.50686 0.13244 0.299(8) 5

S10. Structural Data for Cu-MAZ under flowing O_2 at 250°C

data_ MAZ_250C

_cell_length_a 18.186(6)

_cell_length_b 18.186(6)

_cell_length_c 7.625(2)

_cell_angle_alpha 90

_cell_angle_beta 90

_cell_angle_gamma 120

_cell_volume 2184.0(15)

_symmetry_space_group_name_H-M P63/mmc

loop_

_symmetry_equiv_pos_as_xyz

'x, y, z '

'-x, -x+y, z+1/2 '

'-x, -y, -z '

'-x, -y, z+1/2 '

'-x+y, -x, -z+1/2 '

'-x+y, -x, z '

'-x+y, y, -z+1/2 '

'-x+y, y, z '

'-y, -x, -z+1/2 '

'-y, -x, z '

'-y, x-y, -z+1/2 '

'-y, x-y, z '

'y, -x+y, -z '

'y, -x+y, z+1/2 '

'y, x, -z '

'y, x, z+1/2 '

'x-y, -y, -z '

'x-y, -y, z+1/2 '

'x-y, x, -z '

'x-y, x, z+1/2 '

'x, y, -z+1/2 '

'-x, -x+y, -z '

'x, x-y, -z+1/2 '

'x, x-y, z '

loop_

_atom_site_label

_atom_site_type_symbol

_atom_site_symmetry_multiplicity

_atom_site_fract_x

_atom_site_fract_y

_atom_site_fract_z

_atom_site_occupancy

_atom_site_B_iso_or_equiv

Si01 Si 12 0.6686(2) 0.5081(2) 0.25 1 3.375405

Si02 Si 24 0.73843(16) 0.64466(14) 0.5464(3) 1 3.375405

O003 O 12 0.7085(4) 0.6145(4) 0.75 1 4.910439

O004 O 12 0.83774(19) 1.6755(4) 0.5094(8) 1 4.910439

O005 O 24 0.6736(3) 0.5629(2) 0.4291(5) 1 4.910439

O006 O 12 0.7235(4) 0.7235(4) 0.5 1 4.910439

O007 O 6 0.5751(3) 0.4249(3) 0.25 1 4.910439

O008 O 6 0.7407(3) 0.4814(6) 0.25 1 4.910439

Cu1 Cu+2 2 0.6666667 0.3333333 0.25 0.099(4) 5

Cu2 Cu+2 12 0.4519(8) 0.5481(8) 0.457(3) 0.070(2) 5

Cu3 Cu+2 12 0.417(2) 0.4971(18) 0.25 0.0526(19) 5

O11 O-2 12 0.5067(15) 0.4933(15) 0.122(4) 0.157(6) 5

S11. Structural Data for Cu-MAZ under flowing O_2 at 275°C

data_ MAZ_275C

_cell_length_a 18.185(5)

_cell_length_b 18.185(5)

_cell_length_c 7.624(2)

_cell_angle_alpha 90

_cell_angle_beta 90

_cell_angle_gamma 120

_cell_volume 2183.5(13)

_symmetry_space_group_name_H-M P63/mmc

loop_

_symmetry_equiv_pos_as_xyz

'x, y, z '

'-x, -x+y, z+1/2 '

'-x, -y, -z '

'-x, -y, z+1/2 '

'-x+y, -x, -z+1/2 '

'-x+y, -x, z '

'-x+y, y, -z+1/2 '

'-x+y, y, z '

'-y, -x, -z+1/2 '

'-y, -x, z '

'-y, x-y, -z+1/2 '

'-y, x-y, z '

'y, -x+y, -z '

'y, -x+y, z+1/2 '

'y, x, -z '

'y, x, z+1/2 '

'x-y, -y, -z '

'x-y, -y, z+1/2 '

'x-y, x, -z '

'x-y, x, z+1/2 '

'x, y, -z+1/2 '

'-x, -x+y, -z '

'x, x-y, -z+1/2 '

'x, x-y, z '

loop_

_atom_site_label

_atom_site_type_symbol

_atom_site_symmetry_multiplicity

_atom_site_fract_x

_atom_site_fract_y

_atom_site_fract_z

_atom_site_occupancy

_atom_site_B_iso_or_equiv

Si01 Si 12 0.6682(2) 0.5077(2) 0.25 1 3.375405

Si02 Si 24 0.73855(15) 0.64442(14) 0.5457(3) 1 3.375405

O003 O 12 0.7096(4) 0.6141(4) 0.75 1 4.910439

O004 O 12 0.83690(19) 1.6738(4) 0.5098(8) 1 4.910439

O005 O 24 0.6739(3) 0.5623(2) 0.4277(5) 1 4.910439

O006 O 12 0.7209(3) 0.7209(3) 0.5 1 4.910439

O007 O 6 0.5754(3) 0.4246(3) 0.25 1 4.910439

O008 O 6 0.7400(3) 0.4799(6) 0.25 1 4.910439

Cu1 Cu+2 2 0.6666667 0.3333333 0.25 0.157(4) 5

Cu2 Cu+2 12 0.4528(8) 0.5472(8) 0.460(3) 0.070(2) 5

Cu3 Cu+2 12 0.409(2) 0.4897(19) 0.25 0.0503(19) 5

O11 O-2 12 0.5071(18) 0.4929(18) 0.130(5) 0.126(6) 5

S12. Supplementary References

[1] “Anomalous dispersion calculations near to and on the long-wavelength side of an absorption edge”, D. T Cromer and D. A. Liberman, Acta Cryst., 1981, A37, 267 – 268.

[2] TOPAS-5 Technical Reference, https://www.topas-academic.net/Technical_Reference.pdf.

[3] “Application of symmetrized harmonics expansion to correction of the preferred orientation effect”, M. Järvinen, J. Appl. Cryst., 1993, 26, 525-531.

[4] “Phenomenological model of anisotropic peak broadening in powder diffraction”, P.W. Stephens, J. Appl. Cryst., 1999, 32, 281-289.
